# Gastro-enteritis outbreak among Nordic patients with psoriasis in a health centre in Gran Canaria, Spain: a cohort study

**DOI:** 10.1186/1471-2334-4-45

**Published:** 2004-10-29

**Authors:** Hanne M Eriksen, Philippe J Guerin, Karin Nygård, Marika Hjertqvist, Birgitta de Jong, Angela MC Rose, Markku Kuusi, Ulrike Durr, AG Rojas, Cato Mør, Preben Aavitsland

**Affiliations:** 1 European Programme for Intervention Epidemiology Training (EPIET), 171 82 Solna, Stocholm, Sweden; 2 Department of infectious disease control, Norwegian Institute of Public Health, Norway, PO Box 4404, Nydalen, 0403 Oslo, Norway; 3 Department of infectious disease control, Swedish Institute for Infectious Disease Control, 171 82 Solna, Stocholm, Sweden; 4 Department of infectious disease control, National Public Health Institute, Finland, Mannerheimintie 166 FIN - 00300 Helsinki, Finland; 5 Department of infectious disease control, Instituto de Salud Carlos III, Sinesio Delgado, 4 al 12, 28029 Madrid, Spain; 6 The Gran Canaria Public Health Authority, Las Palmas, Spain; 7 Department of skin disease, Rikshospitalet, Sognsvannsveien 20, 0027 Oslo, Norway

## Abstract

**Background:**

Between November 2 and 10, 2002 several patients with psoriasis and personnel staying in the health centre in Gran Canaria, Spain fell ill with diarrhoea, vomiting or both. Patient original came from Norway, Sweden and Finland. The patient group was scheduled to stay until 8 November. A new group of patients were due to arrive from 7 November.

**Methods:**

A retrospective cohort study was conducted to assess the extent of the outbreak, to identify the source and mode of transmission and to prevent similar problems in the following group.

**Results:**

Altogether 41% (48/116) of persons staying at the centre fell ill. Norovirus infection was suspected based on clinical presentations and the fact that no bacteria were identified. Kaplan criteria were met. Five persons in this outbreak were hospitalised and the mean duration of diarrhoea was 3 days. The consequences of the illness were more severe compared to many other norovirus outbreaks, possibly because many of the cases suffered from chronic diseases and were treated with drugs reported to affect the immunity (methotrexate or steroids).

During the two first days of the outbreak, the attack rate was higher in residents who had consumed dried fruit (adjusted RR = 3.1; 95% CI: 1.4–7.1) and strawberry jam (adjusted RR = 1.9; 95% CI: 0.9–4.1) than those who did not. In the following days, no association was found. The investigation suggests two modes of transmission: a common source for those who fell ill during the two first days of the outbreak and thereafter mainly person to person transmission. This is supported by a lower risk associated with the two food items at the end of the outbreak.

**Conclusions:**

We believe that the food items were contaminated by foodhandlers who reported sick before the outbreak started. Control measures were successfully implemented; food buffets were banned, strict hygiene measures were implemented and sick personnel stayed at home >48 hours after last symptoms.

## Background

On 5 November 2002, the Department of Infectious Disease Epidemiology at the Norwegian Institute of Public Health (NIPH) was contacted by a dermatologist at Rikshospitalet, a tertiary care university hospital in Oslo, Norway. He reported several cases of gastro-enteritis in a medical care centre in Argúinegúin, Gran Canaria, Spain where Nordic patients with psoriasis undergo climate therapy. The symptoms (diarrhoea and vomiting) were reported to be of short duration (1–2 days). The patient group was scheduled to stay until 8 November. A new group of patients were due to arrive from 7 November. An outbreak management team was created in order to investigate and control the current outbreak, and to prevent similar problems in the following group. The outbreak management team consisted of investigators working in public health institutes in the countries involved (Sweden, Finland, Norway and Spain). The investigation was organised and co-ordinated from the NIPH in Oslo, Norway.

The objectives of this investigation were to assess the extent of the outbreak, identify the mode of transmission, the vehicle and the causative pathogen and recommend appropriate control measures.

## Methods

The health centre is a private institution owned by the Norwegian Asthma and Allergy Association. Every three weeks the health centre receives a group of 100–110 Nordic patients suffering from skin diseases (mainly psoriasis). Rikshospitalet, Oslo, Norway administers the project. Patients are residents of Norway, Sweden and Finland. On site, Nordic medical staff (one dermatologist, three nurses and a sport and leisure leader) provides health services for the patients. The centre also hosts private individuals and employees of the Asthma and Allergy Association.

We conducted a retrospective cohort study among all Nordic patients attending dermatological care and employees staying at the health centre between 2 and 10 November. The persons from the Asthma and Allergy Association, and employees eating and staying outside the centre in the same time period were not included since there were no reported cases among this group and they did not eat or use the same facilities as the dermatological patients.

### Case definition

A case was defined as a person who 1) attended dermatological care or worked at the Health Centre in Gran Canaria, 2) took meals at the Centre and 3) fell ill between the 2 and 10 November 2002, with symptoms of diarrhoea (3 or more loose stools in 24 hours), vomiting or both.

### Recruitment of the cohort

Health personnel at the health centre provided a guest list with name, address, age, sex and bungalow number. For cases they also recorded date of onset of symptoms and stool sampling and results of microbiological analysis.

A standard questionnaire was developed at the Norwegian Institute of Public Health, translated in each national centre and mailed to the entire cohort, with instructions to fill it in and send it back to the public health institute in their country of origin.

The local health authorities in Gran Canaria enquired about gastro-intestinal illness in the community. They contacted health centres, churches and schools in the area.

### Exposure

An exposure was defined as a food item eaten or an environmental factor present within the 2 days before onset of illness (the number of cases and the number of persons in the denominator therefore varies for different days of analysis). Possible exposures included consumption of all food items served in the health centre's restaurant, consumption of food served outside the centre, brushing teeth in tap water, drinking tap water, consumption of ice cubes, swimming in one of the two pools, swimming in the ocean, having had contact with an ill person, hand washing habits before meals, and the sharing of bungalow with symptomatic persons. We recorded the number of times food items were eaten to enable the study of potential dose-response relationships.

### Analysis

Day by day, we compared food-specific attack rates (AR) for each item for the exposed and the non-exposed. Suspecting the etiological agent to be norovirus and knowing it has a short incubation period (24–48 hours) [1] only food consumption in the two days preceding onset of symptoms were considered as possible vehicle of the infection. Therefore, when considering food eaten on 31 October, only cases that became ill 2 November were in the numerator. Cases ill on or after 3 November were included in the denominator. When considering food eaten on 1 November, cases that became ill on 2 and 3 November were in the numerator. Likewise, persons were removed from the data set after they were reported ill, since they were no longer at risk. Then we pooled the results for the two first days of the outbreak, assuming that person-to person transmission would be low these first days.

We used EpiData software (Epidata Association, Denmark) for data collection and analysed them with SPSS version 10.0 (SPSS Inc. Chicago, Illinois). Attack rates, relative risk and 95% confidence intervals were calculated for each of the food items and other exposures. Only variables with RR greater than 2.0 are presented in this report. A multivariable analysis was run to assess potential confounding, including age, gender and the pooled variables with RR greater than 2.0 in the univariable analysis.

### Laboratory and environmental investigations

All cases in the cohort presenting gastro-enteric symptoms were encouraged to deliver a stool sample. The samples were sent to a local private laboratory in Gran Canaria. Both virological and bacterial tests were requested.

Food sampling of some of the served items was performed on 5, 21 and 22 November by the local health authorities in Gran Canaria. The health authorities also took samples on 12 November from water taps and from the pools. Both stool and environmental samples were to be sent to Madrid for virology testing. Due to misunderstandings the samples were never forwarded to Madrid. Virological analyses were therefore not performed.

## Results

We mailed 110 questionnaires to the patients who stayed at the Centre between 17 October and 8 November 2002, and employees (n = 6) eating at the centre. Ninety-one questionnaires (response rate = 78%) were returned (2 from employees, 89 from patients at the centre).

### Personal characteristics

Among the 91 respondents there were 47 men and 44 women. The median age was 48 years (range 18–80).

Forty-eight persons fulfilled the case definition (attack rate (AR) = 53%). The AR was not significantly different by genders or nationalities. The AR was higher among those above 70 years of age (80%).

### Temporal distribution

**Figure 1:**
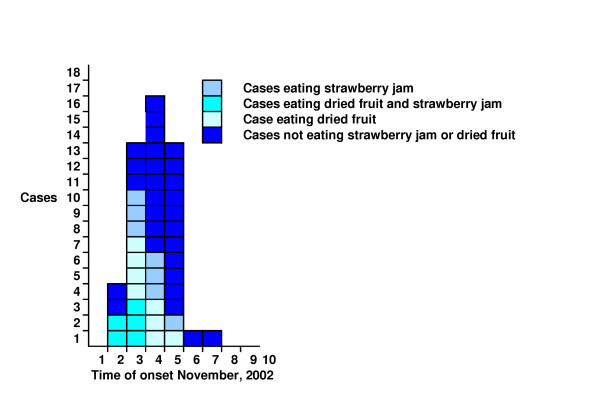
Gastro-enteritis cases (n = 48) by date of onset and exposure to dried fruit and strawberry jam among Nordic patients and employees (n = 91) in a health centre in Gran Canaria, Spain, October-November 2002.

The outbreak peaked on November 4, with 16 cases (Figure 1). The outbreak extended from 2 to 7 November. In addition, two kitchen workers, without dates of onset, were ill with diarrhoea just before the outbreak started. None of the other staff eating outside the centre were reported ill.

No cases were reported among private individuals and employees of the Asthma and Allergy Association staying in the same compound, but who ate only outside the health centre.

### Disease characteristics

Forty-two cases had diarrhoea and 33 vomited (Table 1). The mean duration of symptoms was 3,7 days for diarrhoea (range 1–23 days) and 1 day for vomiting. Twenty-four cases reported having seen a doctor in Gran Canaria. Seven consulted a doctor after returning home. Five persons went to hospital and 11 received intravenous treatment. Six had to stay home from work on average for 1 day. Ten of the respondents reported 24 possible secondary cases among persons they had been in contact with after they returned home.

Cases were distributed among most of the bungalows. The AR for those sharing room with two others were 42% (39/93), AR for those sharing room with one person 38% (3/8) and AR among those 9 living in a single room was 44%. Two cases were employees living private or in a separate section. Six cases fell ill subsequent (more than 10 hours later and within two days) to their roommate.

There were no gastro-enteritis cases reported in the community concurrently to the outbreak in the health centre.

### Cohort study, food specific attack rates

From the daily analysis of food consumption, strawberry jam, dried fruit (eaten on both 1 and 2 November) and butter (eaten on 31 October) were associated with risk of illness (RR> 2.0) for those who became ill on 2 and 3 November (Tables 3 and 4). For those ill 4 and 5 November eating pear was associated with risk of illness (RR 2.8, 95% CI: 0.6–14.4) and eating strawberry jam (RR 1.2, 95% CI: 0.4–3.5). None of these items were independent associated with disease onset 4 and 5 November, in the multivariable analysis.

When pooling food consumption 1 and 2 November, eating pear and drinking full milk also increased the risk of developing gastro-enteritis. In the multivariable analysis including sex, age group, and all variables with RR>2.0, consumption of dried fruit (adjusted RR = 3.1, 95% CI: 1.4–7.1) and strawberry jam (adjusted RR = 1.9, 95% CI: 0.9–4.1) were independently associated with disease (Table 3).

AR for those with who became ill on November 2 and 3 increased with the amount of strawberry jam eaten. No dose response was observed for dried fruits (Table 4).

### Laboratory results and aetiology

We reviewed the clinical symptoms: 69% vomited, 85% had < 72 h duration of illness and all stool samples were found negative for bacteria. Based on this, norovirus was suspected as the aetiological agent. This hypothesis is supported by Kaplan criteria (Table 2) [2].

Stool samples from 6, non symptomatic food handlers were taken on 8 and 12 November and repeated on 28 November. Two of the food handlers were reported to be symptomatic just prior to the outbreak.

All stool samples taken from employees and patients staying at the centre (altogether 43 stool samples) were reported negative for bacteria at the private laboratory in Gran Canaria. Virological analyses were not performed.

Six food items and water from the two pools were analysed for bacteria: chicken croquettes, frozen chicken breast and leg, sausage, cheese and salmon. Results were negative.

## Discussion

We initiated an epidemiological investigation of an outbreak of gastro-enteritis in a health centre in Gran Canaria, Spain. Based on Kaplan criteria, our findings suggest that the aetiological agent was norovirus. Results of the cohort study suggests that the outbreak was initiated by ingestion of dried fruits, strawberry jam or both, followed by person-to-person transmission. There is no supporting microbiological or environmental evidence.

### Pathogenesis and mode of transmission

Norovirus outbreaks can often be diagnosed presumptively on clinical grounds from their characteristic epidemiological features [2]. Kaplan has reported four criteria that indicate with a high sensitivity and a relatively high specificity that a gastroenteritis outbreak is caused by norovirus [3]. In this outbreak all four criteria were met. The Kaplan criteria were used since a confirmatory microbiological diagnosis was pending. We collected time of onset of symptoms, but not of recovery. We therfore had to use 0–72 h cut off instead of 12–60 as in the Kaplan criteria. We do not belive this affected our results.

A high proportion of persons in this outbreak reported that they received IV-treatment (n = 11, saw a general practitioner (n = 24), were hospitalised (n = 5) and the mean duration of diarrhoea was 3.7 days. The reported consequences, especially the duration of the illness was severe compared to many other norovirus outbreaks [3-5]. The explanation may be that many of the cases suffered from chronic diseases and were treated with drugs reported to affect the immunity (methotrexate or steroids). This explanation is supported by two recent reports where norovirus gastroenteritis is described not to be so mild in certain groups in the community [6] and in hospital patients' [7].

We believe there were two modes of transmission: a common source for those who fell ill on 2 and 3 November and thereafter mainly person to person transmission. Low RR for all food items at the end of the outbreak and the short incubation period for noroviruses, together with the fact that norovirus outbreaks usually have high rates of person-to-person transmission [8], support this hypothesis.

We did however not find a higher attack rate among those who shared room or bungalow. Only nine persons had a single room.

There were no cases among those persons who stayed at the health centre, but did not eat in the health centre restaurant or in the community during the outbreak. This suggests a foodborne outbreak with its origin at the centre's restaurant.

### Vehicles of contamination

Dried fruits, strawberry jam or both were the probable vehicles of contamination. Strawberry jam and dried fruits were handled and kept at the kitchen in the Health Centre and not supplied by the catering service. They were served in buffet style on plates. Both food items are biologically plausible vehicles. Contaminated hands or silverware could explain contamination of these food items, but these modes of transmission were not verified. One hypothesis was that the food items were contaminated by the foodhandlers who reported being ill before the outbreak started. Both foodhandlers were involved in preparing the food for the buffet. Very few organisms of norovirus are needed to transmit the disease [4]. The jam was commercial and cooked. To our knowledge, none of these specific food items has been incriminated as a vehicle in norovirus outbreaks reported in the literature. There are, however, several similar food products that have been involved in norovirus outbreaks [1,8,9].

Dose-response analyses give further support for contamination of the strawberry jam. Those who ate jam twice doubled their risk of developing gastro-enteritis.

## Method

Suspecting norovirus, with a short incubation period, as the causal agent [1], we treated each day as a new cohort. We assumed that persons falling ill at the beginning of the outbreak were infected by a common source, while those persons falling ill later could have been infected by person-to-person transmission, by a common source, or both.

When looking at the whole period as a cohort and thus looking at all persons falling ill between 2 and 10 November, then, none of the exposures seemed to increase the risk of disease. The association between food items and disease was probably masked by a high number of cases infected by person-to-person transmission.

Eight cases had not eaten dried fruit during the 2 days before falling ill. The concept of dried fruit is different in Spain from Scandinavia. In Spain, dried fruit means raisins, which were served in the restaurant. In the Nordic countries, dried fruit is in general understood to be a mixture of different types of dried fruits. This difference in concepts may have introduced an information bias.

We asked for food history for 5 days. The food was always served buffet-style. The menus included more than 300 different food items. To reduce potential recall bias, we pooled the food items consumed on either of the 2 days prior to onset. The fact that strawberry jam and dried fruit remained associated for those with onset 2 and 3 November (Table 3) is an argument against the problems related to recall bias.

### Intervention

Based on clinical suspicions of norovirus infection, NIPH suggested to the centre medical personnel to apply a guideline on control of norovirus infection in hospital care setting [10]. We recommended implementing these guidelines with a special focus on improved hygiene measures and individually served food instead of buffet meals. The health personnel at the health centre supervised the implementation of the guideline.

We recommended taking new stool samples of the kitchen workers that had been ill with gastro-enteritis symptoms and excluding all symptomatic food handlers from work for 48 hour after their first normal stool. We also recommended looking for structural and operational deficiencies in the health centre kitchen and in the catering company.

Further environmental investigation by the local public health authorities was recommended. The importance of taking stool samples, and analysing them for both virus and bacterial pathogens, was emphasised.

The control measures were successfully implemented. Guidelines to control norovirus for a hospital care setting are more demanding in hygiene measures than guidelines for hotel outbreaks. Taking into account that qualified health personnel were in place, potential cases were more susceptible to the disease because of underlying diseases, so these strict measures were justified.

It was not possible to cancel the planned arrival of the persons due at the centre from 7 November. Stopping the group of patients with scheduled arrival in the end of November was discussed. Patients expecting to travel to Gran Canaria got written information about the situation at the centre. As no new cases were reported after 14 November (see epilogue), we recommended that this group should travel as planned.

### Epilogue

Among persons arriving 7 November (n = 100), 18 became ill with gastro-enteritis. There were no cases after 14 November. Four of seven stool samples were positive for Salmonella. This finding did not change the belief that norovirus caused the gastro-enteritis among persons in the outbreak described in this article. *Salmonella *was not found in any of the 22 stool-samples from our cohort. This, together with the reported symptoms, suggests that there may have been two different outbreaks at the centre during this period.

## Conclusion

Between 2 and 7 November, 66 persons fell ill with diarrhoea, vomiting or both in a health centre for Nordic patients with skin diseases at Gran Canaria. Our data suggest that norovirus caused the outbreak.

Our findings suggest that individuals who consumed dried fruit (adjusted RR = 3.1, 95% CI: 1.4–7.1) or strawberry jam (adjusted RR = 1.9, 95% CI: 0.9–4.1) were more likely to contract the disease. One hypothesis is that the food items were contaminated by foodhandlers that had had gastro-enteritis shortly before the outbreak started.

Improved hygiene measures and individually served food were successfully implemented.

## Abbreviations

Norwegian Institute of Public Health; NIPH

Attack rate; AR

Relative risk RR

## Competing interests

The author(s) declare that they have no competing interests.

## Authors' contributions

HME: In charge of the investigation, data handeling and writing of the article

PJG: In the outbreak management team, contributed in writing and distribution of the questionaire, and review and comment on the different versions of the article.

KN: In the outbreak management team, contributed in writing and distribution of the questionaire, and review and comment on the different versions of the article.

MH: In the outbreak management team, contributed in writing and distribution of the questionaire, and review and comment on the different versions of the article.

BdJ: In the outbreak management team, contributed in writing and distribution of the questionaire, and review and comment on the different versions of the article.

AMCR: In the outbreak management team, contributed in writing and distribution of the questionaire, and review and comment on the different versions of the article.

MK: In the outbreak management team, contributed in writing and distribution of the questionaire, and review and comment on the different versions of the article.

UD: In the outbreak management team, contributed in writing and distribution of the questionaire, and review and comment on the different versions of the article.

AGR: Working locally with the outbreak, contributed in writing and distribution of the questionaire, and review and comment on the different versions of the article.

CM: In the outbreak management team, contributed in writing and distribution of the questionaire, and review and comment on the different versions of the article.

PA: In the outbreak management team, contributed in writing and distribution of the questionaire, and review and comment on the different versions of the article.

## Pre-publication history

The pre-publication history for this paper can be accessed here:



## Figures and Tables

**Table 1:** Clinical signs and symptoms among the 48 cases and symptoms in non-cases among Nordic patients and employees at a health centre (n = 91) in Gran Canaria, Spain, October – November, 2002.

Symptoms/attribute	Number (percent of all cases)	Median duration of symptoms in cases	Number of non cases with symptoms
Diarrhoea	42 (88)	3 days	2
Blood seen in stool	2 (5*)		0
Vomiting	33 (69)	1 day	0
Nausea	34 (71)	2 days	9
Feeling feverish	12 (25)		3
Abdominal cramps	34 (71)	3 days	4
Headaches	14 (29)	2.5 day	3
General practition visit	24 (50)		1
Stool samples obtained	22 (6 of these after returning home)		0
Intravenous treatment	11 (23)		_
Ambulatory visit in hospital	5 (10)		_
Home from work	6 (13)	1 day	_

• Denominator is only those with diarrhoea (n = 42)

**Table 2:** Kaplan's criteria (2) for suspecting an outbreak is due to norovirus compared to features of the gastro-enteritis outbreak in Gran Canaria 2002.

Kaplan criteria	How our study meet these criteria
1. Vomiting in > 50% of cases	69% vomited
2. Duration of illness 12–60 h	85% had duration of illness -1–72* h
3. Incubation period of 15–36 h	Food items eaten within 48 h are possible vehicles
4. Bacterial pathogens are not identified	22 stool samples found negative

* Duration of symptoms was asked for in terms of days. Only time of onset and not hourly information on recovery were collected. We therefore had to use 1–72 h instead of 12–60 h.

**Table 3:** Food specific attack rates (AR) (uni – and multivariable analysis¤) for pooled food items (food pooled from October 31-November 2) among Nordic patients and workers ill on 2 and 3 November, 2002 at the health centre (n = 91) in Gran Canaria, Spain.

**Food item**	**Food eaten**			**Food not eaten**			**Univariable**			**Multivariable**	
	
	Cases N = 17	Total*	AR%	Cases	Total*	AR%	RR	95% C.I	% cases exposed	RR	95% C.I
**Strawberry jam**	8	24	33	9	63	14	2.3	1.0–5.3	47	1.9	0.9–4.1
**Butter**	7	28	25	10	59	17	1.5	0.6–3.5	41		
**Dried fruit**	9	21	43	8	66	12	3.5	1.6–8.0	53	3.1	1.4–7.1
**Apple**	5	25	20	12	62	19	1.0	0.4–2.6	29		
**Pear**	6	17	35	11	70	16	2.3	1.0–5.2	35		
**Full milk**	4	10	40	13	77	17	2.4	1.0–5.7	24		
**Kiwi**	5	21	24	12	66	18	1.3	0.5–3.3	29		

4 responders did not remember what they ate

¤ Variables controlled for in the multivariable analysis: age, gender and the pooled variables with RR greater than 2.0 in the univariable analysis.

**Table 4:** Attack rates of gastroenteritis by amount of food consumed by Nordic patients and workers with onset on 2 and 3 November, 2002 at the health centre (n = 91) in Gran Canaria, Spain.

Food item and dose	Ill	Not ill.	AR	RR (95%CI)	X^2^_trend_
Dried fruit, not eaten	8	58	12	Ref	
Dried fruit, eaten once	3	4	43	3.5 (1.2–10.4)	
Dried fruit, eaten twice or more	6	8	43	3.5 (1.5–8.6)	
Strawberry jam, not eaten	9	57	14	Ref.	
Strawberry jam, eaten once	2	6	25	1.8 (0.5–7.0)	
Strawberry jam, eaten twice or more	6	8	43	3.1 (1.3–7.4)	6.46 (p = 0.01)
